# In Vitro Degradability, Microstructural Evaluation, and Biocompatibility of Zn-Ti-Cu-Ca-P Alloy

**DOI:** 10.3390/nano12081357

**Published:** 2022-04-15

**Authors:** Navaneethakrishnan Gopal, Parameswaran Palaniyandi, Palanisamy Ramasamy, Hitesh Panchal, Ahmed Mohamed Mahmoud Ibrahim, Mohammad S. Alsoufi, Ammar H. Elsheikh

**Affiliations:** 1 Department of Mechanical Engineering, K. Ramakrishnan College of Technology, Tiruchirappalli 621112, India; gnkrishna08@gmail.com (N.G.); parameswaranp.mech@krct.ac.in (P.P.); 2 Department of Electrical and Electronics Engineering, SRM Institute of Science and Technology, Chennai 603203, India; krspalani@gmail.com; 3 Mechanical Engineering Department, Government Engineering College, Patan, Gujarat 384265, India; engineerhitesh2000@gmail.com; 4 Production Engineering and Mechanical Design Department, Faculty of Engineering, Minia University, Minya 61519, Egypt; ahmedkhalifa@mu.edu.eg; 5 Mechanical Engineering Department, College of Engineering and Islamic Architecture, Umm Al-Qura University, Makkah 24382, Saudi Arabia; mssoufi@uqu.edu.sa; 6 Production Engineering and Mechanical Design Department, Faculty of Engineering, Tanta University, Tanta 31733, Egypt

**Keywords:** microstructure, cytocompatibility, cell viability, biodegradation, corrosion, Zn-Ti

## Abstract

According to the modern era, zinc is one of the best replacements for human bio-implants due to its acceptable degradation, nominal degradable rate, and biocompatibility. However, alloying zinc with other nutrient metals is mandatory to improve the mechanical properties. In this research, Zn-4Ti-4Cu was alloyed with calcium and phosphorous through a powder metallurgical process to make guided bone regeneration (GBR). First, the sintering temperature of the alloy was found with the usage of thermogravimetric analysis (TGA). Tensile and compression tests showed the suitability of the alloy in strength. The microstructural characteristics were provided with EDS and SEM. The different phases of the alloy were detected with X-ray diffraction (XRD). We can clearly depict the precipitates formed and the strengthening mechanism due to titanium addition. An electrochemical corrosion (ECM) test was carried out with simulated body fluid (Hank’s solution) as the electrolyte. Cytotoxicity, biocompatibility, mechanical properties, and corrosion resistance properties were studied and discussed.

## 1. Introduction

In the 1980s, heat stents were introduced for coronary artery disease, which causes the narrowing and shrinking of the coronary arteries. This disease is caused due to the accumulation of plague/fats and cholesterol, which causes a clot in the valves leading to, in the worst cases, death. The stent was made up of a bare-metal stent (BMS), including 316 stainless steels and cobalt–chromium alloys [1]. The problem with the BMS was restenosis which needed further treatment, such as long-term anti-thrombotic medication administered orally and angioplasty, and thus ended in failure. To replace this, drug-eluting stents were introduced, which have a thin layer of plastics containing drugs (anti-proliferative). The drawback was plastics usage for prolonged drug delivery [2]. The worst problem of the above two technologies of heart stenting is that they are not compatible with infants and children [3].

Fe biomaterials have had a higher corrosion resistance for the past three decades, and Mg has a lower resistance to corrosion in the human body. Therefore, we have to develop an alternative material that should be biocompatible, have higher strength, and higher corrosion resistance [4]. In this case, many researchers chose Zn to suit the need for a bioabsorbable material for cardiovascular stents. Furthermore, it has properties such as superior mechanical properties with reasonable degradation rates in human implants, biocompatibility, etc., making it a suitable material for this generation [5]. H.R. Bakhsheshi-Rad et al. [6] found that a Zn-0.5Al-0.5Mg alloy presents good biocompatibility and prohibits the antibacterial (Escherichia coli) activity. The corrosion rate increased with the addition of Mg, and the cell viability increased up to 110% in the Zn-0.5Al alloy with MC3T3-E1 cells.

When Ca (nutrient element) was added to the Zn-1.5Mg, it possessed excellent strength. During the corrosion of Zn, calcium phosphate precipitation occurs from the surrounding fluid [7,8]. With Ca, Mg and Sr should also be added, which paves the way for newer research ideas on biomaterials. These materials are associated with new bone formation and have higher compressive strength than pure Zn. [9]. When Zn was alloyed with copper (Cu), its mechanical properties, especially tensile strength and percentage of elongation, were improved drastically. This property was much needed for cardiovascular stent application.

Increasing the amount of Cu added, enhances the mechanical and biocompatible properties, but its corrosion rate was a little higher when compared with pure Zn. In addition, due to Cu inclusion, the antibacterial property of the alloy improved. The corrosion rate in the SBF (simulated body fluid) was 22 to 33 μm/year [10]. Hongtao Yang et al. [11] designed a binary Zn alloy, with the inclusions of Mg, Ca, Sr, Li, Mn, Fe, Cu, and Ag, and found that Zn-Li was the most promising alloy for bone replacement due to its higher strength and biocompatibility. They suggested that Zn alloys can have properties close to those of a pure Ti implant in human bio-implants.

Shi [12] fabricated biocompatible material with Zn-0.8Mn with Ag, Ca, or Cu additions to improve strength and cytotoxicity properties. The results found that, with the addition of Ca to Zn-0.8 M, the material provides good strength, and with Cu, the material has good antibacterial activity and ductility. Finally, they suggested that Zn-0.8Mn-0.4Cu offers the best comprehensive properties. M. Bobby Kannan et al. [13] designed a new Zn alloy material Zn-5Al-4Mg and checked its in vitro degradation behavior and biocompatibility using human alveolar lung epithelial cells (A549) and found that the alloy exhibited lower density and higher hardness. The alloy can be used as human mini-orthopedic implants. Mostaed et al. [14] developed four binary alloys, initially, through casting hot extrusions to produce a stent material with a 4mm outside diameter. The Zn-0.5Mg binary alloy showed higher mechanical and corrosion properties with slower degradation rates in Hank’s solution.

Guo et al. [15] researched porous zinc membranes with 300 μm and 1000 μm for guided bone regeneration (GBR). The study shows that the zinc membrane with 300 μm pores exhibited a better choice for the implants. It possessed a valid degradation rate in vitro and higher mechanical properties. Zinc is the essential element in the human body for signal transduction, apoptosis regulation, growth, mineralization of bone tissues, stimulation of cellular proteins, nucleic acid metabolism, initiation resorption, and also helps in maintaining the bone mass [16,17]. Zhang et al. [18] conducted research on a Zn-2Cu alloy with an addition of titanium (Ti), and investigated its microstructure, mechanical, and biodegradation properties, and found that two phases formed CuZn_5_ and newly formed TiZn_16_, which are responsible for better mechanical properties and lower degradation rates. The corrosion resistance is thus enhanced with the addition of Ti (biocompatible) in the matrix.

When Ti is added to the Zn matrix, it forms an intermetallic phase of Zn-Ti, increasing the mechanical properties by up to 4%. This alloy can be applied in human cardiovascular bioabsorbable stents [19]. Lin et al. [20] developed a Zn-1Cu-0.1Ti biodegradable alloy with antibacterial properties for orthopedic implants. The alloy was fabricated with hot and cold rolling. It was finally concluded that the alloy was to be used in bone screws and plates. The fabricated biomaterials need to be porous to interact for bone ingrowth and facilitate cell infiltration. The porous Zn scaffolds can effectively fight and kill bacteria (antibacterial property). Therefore, it suits bone implantation with bioresorbable properties [21]. However, they used the FDM technique for creating porous surfaces. Porous zinc was prepared through spark plasma sintering with different sizes of metal powders. The developed Zn material has the properties of lower degradations, lower ion releases, and optimum compressive properties similar to that of human trabecular bone [22,23]. The quaternary alloy Mg-Zn-Y-Nd, with lower hardness and inhibition properties towards the cancer pathological cells, has been implemented for bio-implants, especially in stent application which helps esophagus cancer patients to easily swallow food and drinks [24]. Ca and P play a significant role in bone regeneration, and their coatings and additions improve osteointegration. The coatings of calcium and phosphorous does two things to improve the degradation rate and decrease it [25,26]. The mechanical alloying method has the advantage of having porous surfaces for GBR and, also, enhances cell attachment. In this research, we have fabricated pentanary Zn-4Ti-4Cu-2Ca-1P as they decrease haemolysis and calcium and phosphorous in bone and implantations. In this work, the considered alloy has to be checked for cytotoxicity, cell viability, etc., taken as per in vitro procedure. Once the alloy suits the in vitro applications, it will be transferred to in vivo processing.

The developed alloy, through the sintering technique, obtains porousness for acceptable guided bone regeneration and degradation rate in in vitro analysis. Although much research was published on zinc as a predominant bio-implant, further investigation into processing is still needed to investigate how the processing affects biocompatibility. In addition, few articles deal with the quaternary alloy formation with Zn, and very few articles on zinc were fabricated with sintering techniques for biomaterial applications.

## 2. Experimental Work

### 2.1. Alloy Design and Sample Preparation

High-purity metal powders (99 to 99.8%), of less than 45 μm, of Zn, Ca, Cu, P, and Ti were imported from Alfa Aesar, Lancashire, UK. As per the compositions presented in Table 1, the metals mentioned above were mechanically alloyed through ball milling using a Fritsch Pulverisette P5 high-energy ball mill, Fritsch Asia–Pacific Pvt. Ltd., Singapore [27,28]. The ball milling was carried out for 8 h using stainless steel vials with a ball/powder weight ratio of 10:1, respectively.

The process controlling agent Toluene was used, which was purchased from Spectrum Chemical Manufacturing Corporation, New Brunswick, NJ, USA. The obtained milled powder was compacted using punch and die, using a universal testing machine following the procedure [29]. The compaction pressure was determined by the following Equation [30]:

(1) p=σsη*(2−R2)[R−R01−R0]nR0(1−R2)(4−R4)

where p is the compacted pressure, R is the relative density, σ_s_ is stress-124 MPa, R_0_ is the relative density of zinc tablets at zero compaction, η* is the efficiency coefficient of a compaction test due to the die friction, and n is the hardening coefficient of the metal powder.

The sintering was carried out in a CO_2_ atmosphere after analysis with TGA (thermo-gravimetric analysis). The sintering temperature was 450 °C by the TGA curve and [31]. The L/D ratio of the element was one. The sintered samples obtained with a diameter of 50 mm and length of 50 mm were polished with 3000 grit sheets. It was then cleaned with acetone and alcohol. The green density and sintered density of the alloy were calculated.

### 2.2. Porosity and Density

The porosity of the samples was determined using the Archimedean method based on water imbibitions. The total volume of each sample was assumed to be a solid cylinder. The equations, formula, and method were taken from [22]:

(2) $$\rho_{\mathrm{por}}=\frac{4\cdot m}{\pi \cdot v\cdot d^{2}}$$

where ρ_por_ is the density of the porous sample, m is its weight, v is its height, d is the diameter of the porous sample, ρ_Zn_ is the density of zinc, and P is the porosity of sample:

(3) $$P=\left(\frac{\rho_{\mathrm{por}}}{\rho_{\mathrm{Zn}}}1\right)\cdot 100\%$$

### 2.3. Mechanical Testing and Microstructure Characterization

Finally, the hardness of the sintered sample was measured by applying a load of 1 kg with a dwell time of 10 s in a Vickers micro-hardness tester (Shimadzu Corporation, Kyoto, Japan). The compression test was taken for five samples at an average according to ASTM E9-89a standards with the INSTRON 5969, USA, machine at room temperature. The compression test was taken from [23]. The test samples were taken with the dimensions of 5 × 5 × 5 mm cube, and an average of five samples were taken. The test was done with a universal testing machine with a strain rate of 10^−3^/s.

The specimens with a cross section of 10 × 10 mm were polished with diamond paste of 0.1 μm. The polished specimens were then cleaned with distilled water. The samples were then etched with 4% nitric acid and alcohol solution. XRD analyses were made using a PANalytical B.V., Almelo, the Netherlands, with Cu kα radiation and a scanning range from 10° to 90° at a scan rate of 2°/min was carried out to identify the phases present in the alloy. The microstructures were examined with SEM (TESCAN VEGA-3).

### 2.4. Electrochemical and Immersion Tests

The electrochemical corrosion testing was conducted at room temperature with Hank’s solution as an electrolyte [9,20] using Biologic instruments-SP 150, Seyssinet-Pariset, France. The sample surface was grinded with 3000 grit sheets, cleaned with ethanol, polished, and dried before taking the test. The sample size for the test was 10 mm in diameter and 2 mm in thickness. The open circuit potential (OCP) of each sample was measured. The electrochemical test included three electrodes: one saturated calomel electrode (SCE), the counter electrode, taken as platinum, and the other, which was the alloy. A rate of 100 mV/min was maintained as the sweep rate for the corrosion process. The polarization curves were obtained using a standard Tafel extrapolation method using ASTM G59-97 [32]. The rate of electrochemical corrosion was measured by Equation (4):

rate of corrosion (mm year −1) = (3.27 × 10^−3^ × i_corr_ × EW)/D (4)

The samples were immersed in SBF solution at 37 °C for 30 days. This was followed by ASTM G31-72 for the immersion test. The pH of the solution was maintained on all the days. The solution was refreshed every 48 h, periodically. SEM analysis was carried out on the corroded area to check the corrosion morphology of the samples. The corrosion rate was calculated by Equation (5), and five samples were tested on average:

C = Δmρ^−1^A^−1^t^−1^ (5)

where C is the corrosion rate, A is the surface area of the sample before testing, and t is the implantation time.

### 2.5. ETBr/AO Staining

For the cell culture, a Vero cell line was used, and it was cultured in a medium of DMEM with 10% fetal bovine serum, 100 u/mL penicillin, and 100 µg/mL streptomycin. It was cultured at 37 °C and in a CO_2_ atmosphere (5%). A total of 5 × 10^5^ cells/mL of Vero cells were plated in a 24-well tissue culture plate and incubated for 24 h in a DMEM growth medium. After incubation, the plate was washed with PBS and treated with a ZCC sample in a serum-free DMEM medium. The plate was incubated at 37 °C in a 5% CO_2_ incubator for 24 h. A total of 50 µL of 1 mg/mL ethidium bromide and acridine orange was added to these wells and mixed gently after incubation. Finally, the plate was centrifuged at 800 rpm for 2 min and evaluated immediately for less than an hour. At least 100 cells were examined by a fluorescence microscope using a fluorescent filter.

### 2.6. MTT Assay

A cytotoxicity test was conducted with the ZCC sample (in vitro). Vero cells was used by 3-(4,5-dimethylthiazol-2-yl)-2,5-diphenyltetrazolium bromide. With a 15 mL culture tube, the Vero cells were harvested. Then, the cells were plated at a density of 1 × 10^5^ cells/mL cells/well (200 µL) into a 96-well tissue culture plate in a DMEM medium containing 10% FBS and 1% antibiotic solution for 24–48 h at 37 °C. These wells were sterilized with PBS and treated with the ZCC samples available at various concentrations in order to make a serum-free medium. The test was conducted thrice at 5% CO_2_ and incubated at 37 °C for 24 h. MTT (20 µL of 5 mg/mL) was added to each well and placed for 2 to 4 h. While visualizing with an inverted microscope, we saw the purple precipitates. It was aspirated off with MTT (220 µL) and washed with 1X PBS (200 µL). In order to dissolve the crystal, a DMSO of 100 µL was added by shaking the plate for 5 min. The absorbance for each well was measured at 570 nm using a microplate reader (Thermo Fisher Scientific, Waltham, MA, USA), and the percentage cell viability and IC50 value were calculated using GraphPad Prism 6.0 software (San José, CA, USA).

## 3. Results

### 3.1. Compaction Pressure and Sintering

The compaction pressure plays a vital role in the green density and porosity of the sample. Compaction pressure was determined using Equation (1) framed by Quadrini et al., an analytical model with the inclusion of particle morphology [30]. The compaction pressure was maintained at 350 MPa to obtain higher strength in the material and to maintain porosity. This will provide the hardness uniformness throughout the sample. The obtained powder will have a lower particle size of 44 microns and, so, the deformation during compaction will be less. However, it will lead to grain boundary strengthening, and the porosity will be reduced [33].

**Figure 1:**
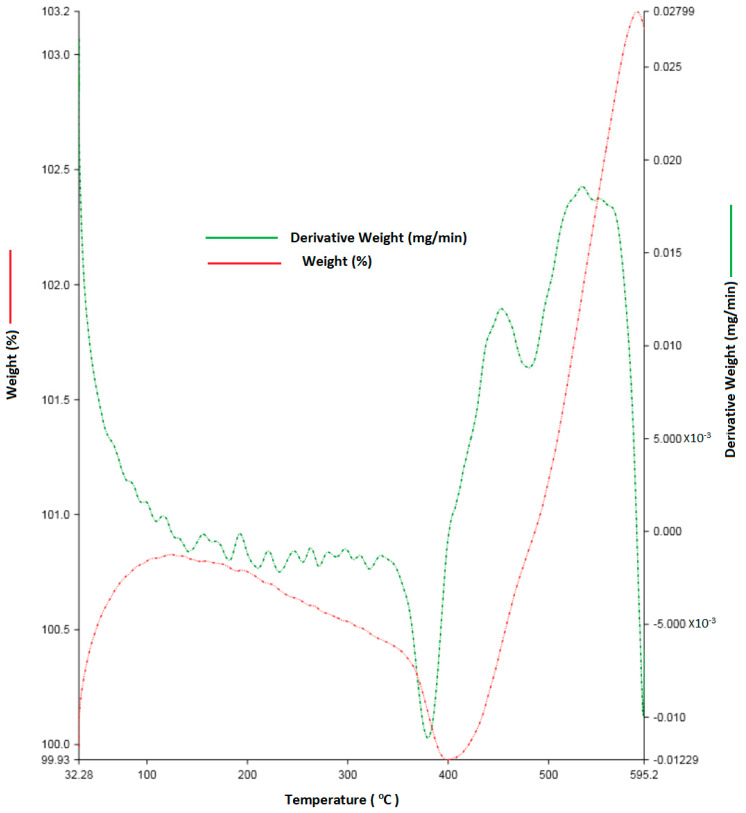
Thermogravimetric analysis of biodegradable Zn alloy.

Figure 1 represents the sintering temperature to bond the metal powder into a biodegradable alloy. Here, the analyzed and fixed temperature is 385 °C for the sintering process. This is compared with [34], in which the nickel was added up to 20% of the weight and where they reached 357 °C. The increase in sintering temperature for the present alloy is due to the addition of the Ti and Cu mixed in the matrix.

### 3.2. Mechanical Properties

The calculated theoretical density of the alloy was 6.25 g/cm^3^, and the actual experimental density was found to be 5.10 g/cm^3^, which was influenced by the compaction pressure and sintering time. The porosity of the sample was 12–17%, on average based on all the samples. The micro-hardness of the sintered samples shows 79.34 ± 3.11 Hv. This hardness value was due to the addition of elements in large quantities. The compressive strength shows 214 ± 24.38 MPa (compressive yield strength) on average of the five samples. Ultimate compressive strength cannot be predicted due to the Zn alloy’s compression super-plastic character, which has an hcp crystal structure [35]. The biodegradable materials should match the mechanical performance of the local bones and tissues for their suitable applications. The gain in mechanical properties was due to the presence of alloying elements.

### 3.3. Composition and Microstructures Characterization

**Figure 2:**
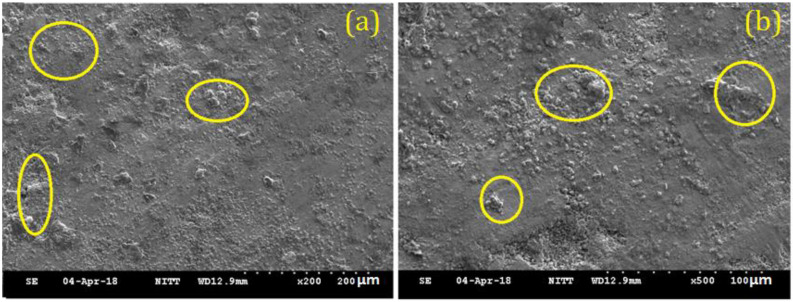
SEM image of the biodegradable zinc alloy (**a**) 200 µm and (**b**) 100 µm.

**Figure 3:**
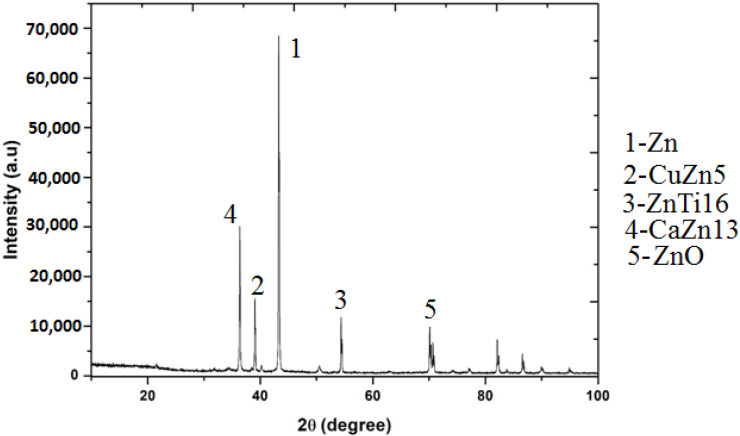
XRD: biodegradable zinc alloy.

**Figure 4:**
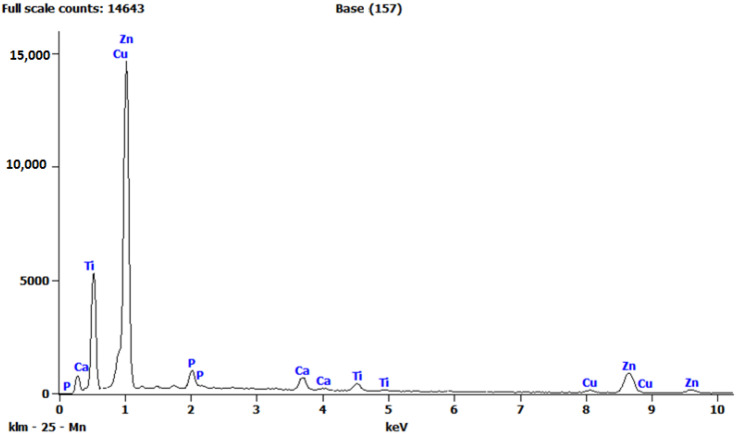
Chemical composition by EDS.

**Figure 5:**
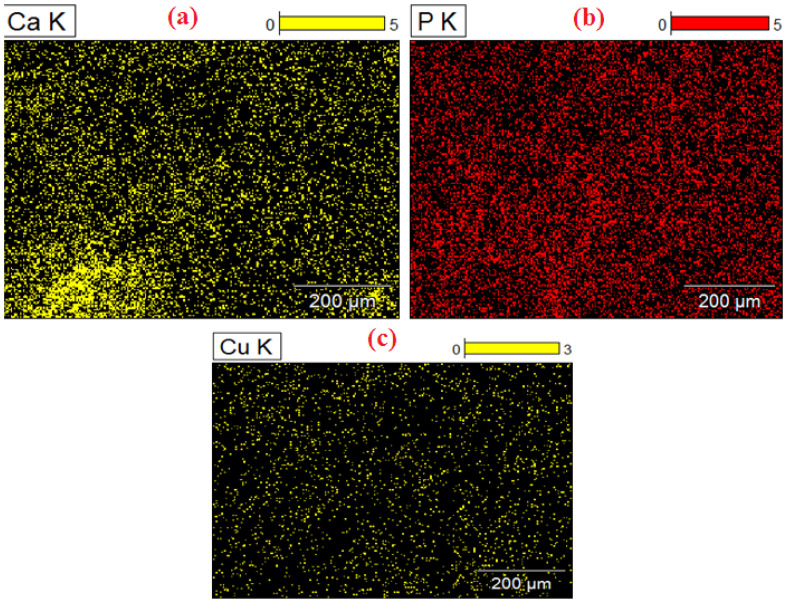
EDS: elemental mapping (**a**) Copper (**b**) Phosphorus (**c**) Calcium.

As the alloys consist of Zn, a hexagonal closed-packed structure was observed throughout the material. The SEM images of the sintered specimens are shown below in Figure 2a and b. The SEM images show porous areas, which help in cell adhesion when implanted. XRD patterns were observed for better characterization, and α-Zn, CuZn_5_, ZnO, ZnTi_16_, and CaZn_13_ are the phases shown in Figure 3. This occurred due to the semi-finished sintered product, and we can predict that the material would have coarse grains. The EDS test confirmed the presence of Zn, Ca, P, Cu, Ti (Figure 4), and the distribution of the alloying elements is shown in the elemental mapping in Figure 5a–c and Table 2.

### 3.4. Corrosion Properties

**Figure 6:**
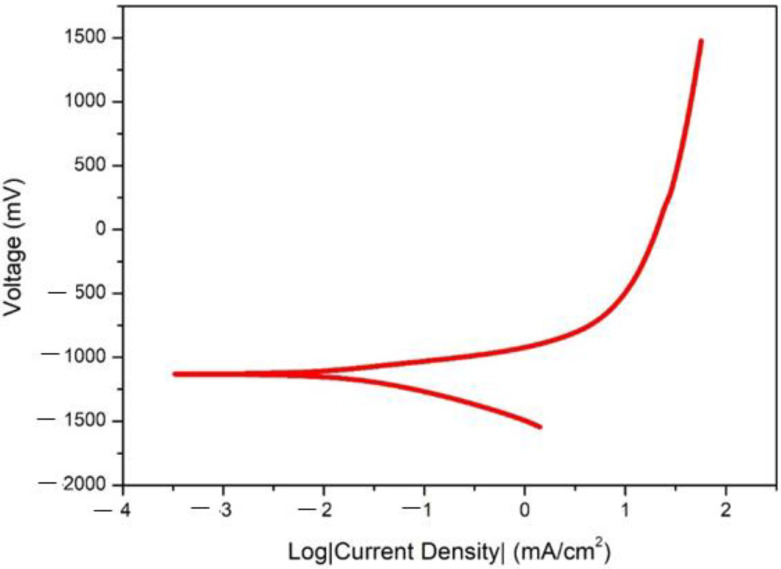
Polarization curve.

The potentiodynamic polarization curve is shown in Figure 6. Hank’s solution was used as an electrolyte. The corrosion potential E_corr_ and current density I_corr_ was obtained from the standard Tafel extrapolation method. On the developed alloy, five tests were conducted for identifying the corrosion rates and this was found based on the average result. The value of E_corr_ was −1.23 (mV) and for I_corr_ was 14.42 mA/cm^2^. We that found the corrosion rate was 0.18 mm/year based on the calculations. We have achieved lesser corrosion rates in the sintering process itself; in other words, without additional mechanical processes, we gained better corrosion results.

### 3.5. Cell Viability

**Figure 7:**
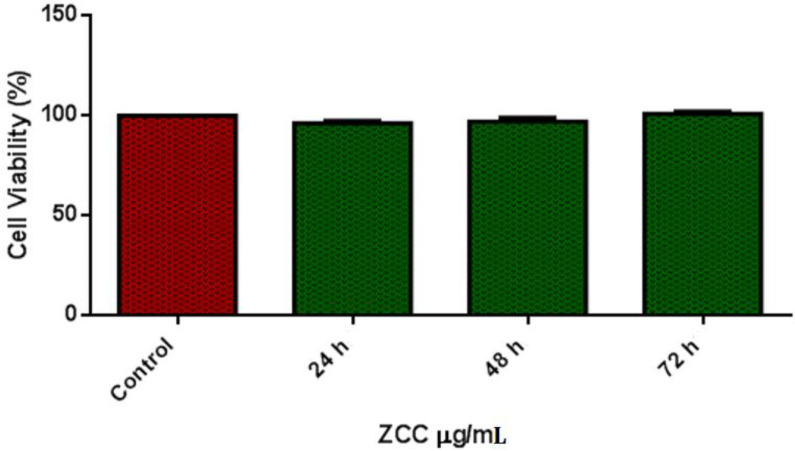
Cell viability–Vero cell line.

The cell viability test was taken with the prepared extracts of the alloys. According to ISO 10993-5, 80 to 100% cell viability is slightly toxic. In addition, according to ISO standards of Part Five (International Organization for Standardization, 1999), cell viability above 75% is considered an acceptable toxicity level. The good cytotoxicity characteristics of the presented alloy material as presented in Table 3. Due to the addition of alloying elements, the level of toxicity was much reduced. All cells cultured show metabolic activities above 90% of the control, showing no cytotoxic in the alloy. Relative proliferation increased when cultured for above 48 h, as shown in Figure 7.

## 4. Discussion

### 4.1. Phase Formation

Ca addition will reduce the dendrite structure and cause grain refinement due to the formation of lower-sized CaZn_13_. It is smaller in size than other particles, such as ZnO. ZnTi_16_ will be attached to it, and the phase ZnTi_16_ is a eutectic structure, and they concentrate on grain boundaries [20]. The addition of both Ti and Cu improves grain refinement and the mechanical properties. The primary intermetallic in the matrix is ZnTi_16_ and CuZn_5_. The elements that cause the grain refinement are Ti and Ca [36]. The ZnTi_16_ phase was discontinuous, and its fiborous structure helps in grain refinement and CuZn_5_. Due to the sintering operation, the specimen will have a coarse grain structure and may have discontinuous phases. The calcium and phosphorous do not form any phases so that they can be moved along the grain boundaries. This may weaken the strength of the material. Both calcium and copper react with zinc, reducing the grain size and forming CaZn_13_, and the reference confirms this [37].

### 4.2. Mechanical Properties

The hardness Vickers test was performed to find out the micro-hardness of the sample. The hardness was obtained as 79.3. This improvement was due to the added alloying elements. Cu addition results in solid-solution strengthening. The phases CuZn_5_ and TiZn_16_ result in enhanced strength by restricting the movement of dislocation as they distribute along the grain boundaries. Due to this, compression properties will increase in the material [10,18]. CuZn_5_ will have a finer dendritic structure, which improves the material’s tensile properties. For cardiovascular stent applications, the design criteria are a yield strength of more than 200 MPa and an elongation of more than 15%. Our developed product meets the criteria. In the compression test, the present alloy showed excellent results and thus is applicable for stent application.

### 4.3. Corrosion Behaviour

**Figure 8:**
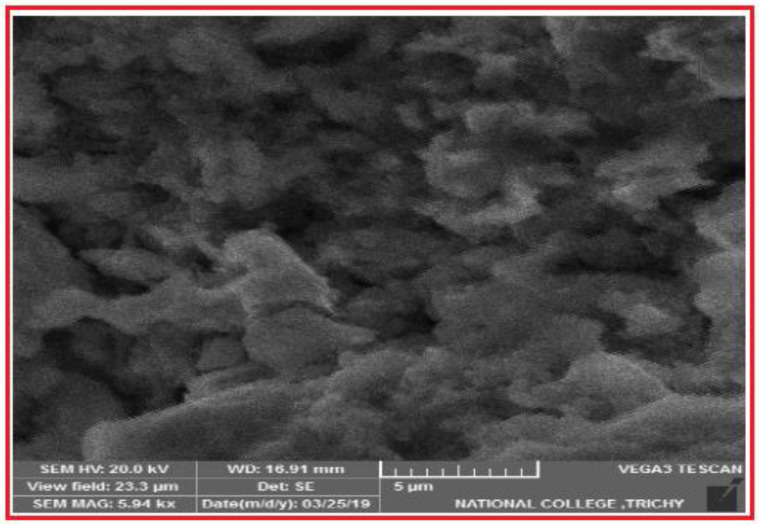
Corroded areas showing many pits.

The alloy exhibits better corrosion resistance properties when compared with all other available alloys. The advantage of this alloy is that it was produced through powder metallurgy, and the specimen was solution treated in order to obtain better properties. The degradation will be Zn + 2H_2_O = Zn^2+^ + H ↑ + 2OH. The formed CuZn_5_ phase increases the corrosion due to galvanic action. Therefore, it weakens the corrosion resistance of the product. Figure 8 shows the galvanic corrosion and its morphologies that occur at the pits formed in the specimen, which is the reason for the increase in the degradation rate. With the addition of Ti, we can expect an increase in corrosion resistance. We can also see there were more pits, due to the Ti addition [18]. The addition of Ca also improves the material’s corrosion resistance [1], but the amount was not drastic. As reported [21], calcium and phosphorous have little effect when used as alloying elements but improve corrosion resistance when coated over zinc. In XRD, there is no evidence of the formation of any phases with calcium and phosphorous. The corrosion products are not harmful and do not cause any irritation, as reported in [10,18,20]. The increased corrosion rate will cause calcium addition [37].

### 4.4. Cell Viability

**Figure 9:**
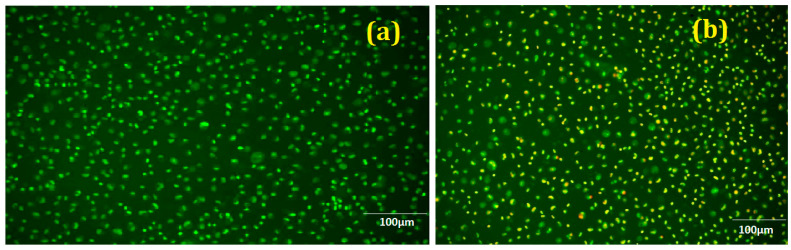
Cell growth and proliferation (**a**) controlled (**b**) treated with ZCC.

**Figure 10:**
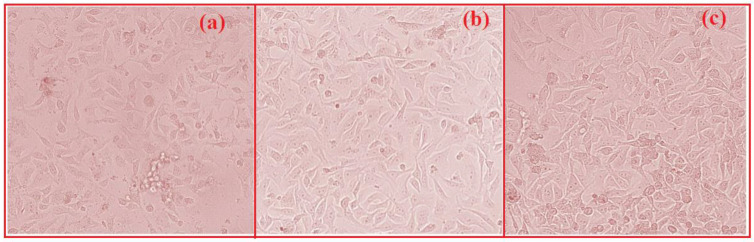
(**a**) controlled, (**b**) ZCC treated cells—24 h, (**c**) ZCC treated cells—48 h.

The designed alloy showed excellent cytotoxic characteristics as cell viability increased above 90% in all the controlled atmospheres. The added alloying materials, Cu, Ti, Ca, and P, have excellent compatibility with the human body. Ti and Cu both have the property of high strength. From Figure 9a and b, we can see the live and dead cells. Cell proliferation was due to the addition of Cu [38]. We can see that there was no notable increase in pH value. We can also see limited dead cells present under fluorescence. The osteogenic activity will be increased due to the release of Zn^2+^ ions when alloyed with Ti [39]. Most of the Ti content will be excreted through urine during degradation, noticed by Woodman et al. [40], so a higher addition of Ti will not affect the body (tested in Vero cells), as presented in Figure 10a–c. The total number of dead cells was found to be less, and we also noted the multiplication of cells.

## 5. Conclusions

In this study, the zinc alloy was designed and prepared by adding the preferred materials, such as copper, titanium, calcium, and phosphorous. The fabricated alloy was tested for strength, degradation behavior, and cytotoxicity. The following findings were completed with the research:

1. Through microstructural studies, it is confirmed that the presence of different phases distributed uniformly and in grain boundaries. The majority of the phases, α-Zn, CuZn_5_, ZnO, ZnTi_16_, and CaZn_13,_ are found through XRD. The mechanical properties are acceptable, and the material has higher strength due to the addition of Ti and Cu in the matrix.
2. The degradation rate was not much more significant than in our previous research [41,42], as the product was made through the sintering and aging process. Better corrosion properties made the alloy suitable for biomedical applications.
3. The cytocompatibility test proves there was cell growth, during the observation, without more cell deaths. Thus, the presented material can be used as a bio-implant. Future work will be done with human cells.

## Figures and Tables

**Table 1:** Chemical composition in weight percentage.

S.NO	Cu	Ti	P	Ca	Zn
1	4	4	2	4	86

**Table 2:** Chemical composition of Zn-Ti-Cu-Ca-P alloy by EDS.

Element Line	Weight %	Weight % Error	Atom %
*P K*	3.86	+/−0.10	7.5
*P L*	---	---	---
*Ca K*	4.39	+/−0.07	6.58
*Ca L*	---	---	---
*Ti K*	3.94	+/−0.16	4.95
*Ti L*	---	---	---
*Cu K*	5.64	+/−0.66	5.34
*Cu L*	---	---	---
*Zn K*	82.18	+/−1.45	75.63
*Zn L*	---	---	---
*Total*	100		100

**Table 3:** Cell viability OD value at 570 nm; control mean OD value: 0.428.

S. No	Tested Sample Concentration (μg/mL)	OD Value at 570 nm (in Triplicates)		Cell Viability (%) (in Triplicates)		Mean Value (%)
1	Control	0.424	0.432	100	100	100
2	24 h	0.416	0.408	97.19	95.32	92.25
3	48 h	0.418	0.422	95.66	98.59	97.12
4	72 h	0.429	0.436	100.23	101.86	101.04

## Data Availability

Not applicable.
